# AChRs Degeneration at NMJ in Aging-Associated Sarcopenia–A Systematic Review

**DOI:** 10.3389/fnagi.2020.597811

**Published:** 2020-12-10

**Authors:** Zhengyuan Bao, Can Cui, Simon Kwoon-Ho Chow, Ling Qin, Ronald Man Yeung Wong, Wing-Hoi Cheung

**Affiliations:** ^1^Department of Orthopaedics and Traumatology, Prince of Wales Hospital, The Chinese University of Hong Kong, Hong Kong, China; ^2^The CUHK-ACC Space Medicine Centre on Health Maintenance of Musculoskeletal System, The Chinese University of Hong Kong Shenzhen Research Institute, Shenzhen, China

**Keywords:** acetylcholine receptors, aging, skeletal muscle, sarcopenia, systematic review

## Abstract

Sarcopenia is an aging process with a decline of skeletal muscle mass and function, which is a challenging public health problem with reduced quality of life in patients. The endplate, the post-synaptic part of the neuromuscular junction (NMJ), occupies 0.1% of the myofiber surface area only, but is composed of millions of acetylcholine receptors (AChRs) that are efficient in binding to acetylcholine (ACh) and triggering skeletal muscle contraction. This systematic review aims to examine aging-associated alterations of post-synaptic AChRs, including morphology, function and related gene expression. A systematic literature search was conducted in PubMed, Embase and Web of Science with relevant keywords by two independent reviewers. Original pre-clinical and clinical studies regarding AChRs changes during aging with available full text and written in English were included. Information was extracted from the included studies for further review. In total, 30 articles were included. Various parameters assessing AChRs alterations by radioassay, immunofluorescence, electrophysiology and mechanical test were reported. Endplate fragmentation and denervation were common in old skeletal muscles during aging. To ensure efficient NMJ transmission and force generation, type I or IIb muscle fibers tended to have increased ACh quanta releasing after electrical stimulations, while type IIa muscle fibers tended to have stronger binding between ACh and AChRs, but the overall function of AChRs was reduced during aging. Alterations of AChRs area depended on muscle type, species and the progress of muscle atrophy and type I muscles fibers tended to demonstrate enlarging AChRs areas. Myogenic regulator factors (MRFs) can regulate the expression of AChRs subunits, while decreased MRF4 may lead to expression changes of AChRs subunits during aging. Sarcoglycan-α can delay low-density lipoprotein receptor-related protein 4 (LRP4) degradation. This protein was increased in old muscles but still cannot suppress the degradation of LRP4. Investigating the role of these AChRs-related genes in the process of aging may provide a potential target to treat sarcopenia.

## Introduction

Sarcopenia is a progressive decline in skeletal muscle mass and function in aging. This is a common cause of reduced physical performance in older people and is coded in the International Classification of Disease, Tenth Revision (ICD-10) Clinical Modification (CM) (Kwon and Yoon, 2017; Wong et al., 2019). According to the latest definition of European Working Group on Sarcopenia in Older People (EWGSOP) in 2019, low muscle mass assessed by dual-energy X-ray absorptiometry (DXA) and strength measured by grip strength will be diagnosed as sarcopenia that can start intervention clinically (Cruz-Jentoft et al., 2019).

The etiology of sarcopenia is multi-factorial, with deterioration of neuromuscular junction (NMJ) as one of the major causes. NMJ serving as the bridge between nervous and muscular system is essential to transmit the action potential from lower motor neurons to induce muscle contraction (Rygiel et al., 2016; Kwon and Yoon, 2017). Aging-associated degeneration of NMJ has been well-documented in several studies (Smith and Chapman, 1987; Mcmullen and Andrade, 2009) and recently it is suggested that NMJ degeneration precedes the appearance of sarcopenia during aging (Deschenes et al., 2010; Tamaki et al., 2014).

NMJ is a complex synapse between motor neurons and muscle fibers, which is composed of three elements: pre-synaptic active zones in motor nerve terminals, intra-synaptic basal lamina and post-synaptic acetylcholine receptors (AChRs) in the muscle membrane (Gonzalez-Freire et al., 2014; Burden et al., 2018). More than 10 million AChRs constitute a specific apparatus named “endplate” occupying ~0.1% of the surface area of a myofiber (Burden et al., 2018; Liu and Chakkalakal, 2018). The post-synaptic AChRs in skeletal muscles are nicotinic and four different types of polypeptide chains compose a heteropentamer: two α subunits and one of each β, γ, and δ subunit (ε subunit will replace the embryonic γ subunit in adults) (Punga and Ruegg, 2012; Liu and Chakkalakal, 2018). As the core element of post-synapse, AChRs' binding with acetylcholine (ACh) is essential for the transition of chemical to electrical signal, eventually leading to muscle contraction.

Morphological changes of NMJ during aging and molecular mechanisms underlying these alterations have been well-summarized before (Tintignac et al., 2015; Larsson et al., 2019), but no review ever focused on aging-related changes of AChRs' function. Besides, considering the important role of NMJ in the progress of sarcopenia and the core role of AChRs at NMJ, these receptors may be one potential therapeutic target for this disease. Hence, a systematic review was conducted to do a comprehensive summary and analysis of aging-associated changes of NMJ post-synaptic AChRs, including morphology, function and gene expression together.

## Methods

### Search Strategy

Literature search was performed on PubMed, Embase and Web of Science (last access to both on 17th Aug 2020). The keywords used were aging, aging, aged, age, acetylcholine receptor^*^, AChR^*^, neuromuscular junction and NMJ. We combined the keywords as (aging OR aging OR aged OR age) AND (acetylcholine receptor^*^ OR AChR^*^) AND (neuromuscular junction OR NMJ) and searched in all fields. This search strategy was used for both databases. PRISMA guidelines were followed.

### Search Criteria

Inclusion criteria were: (1) pre-clinical or clinical studies related to morphological and functional changes in NMJ during aging by evaluations of the AChRs; (2) studies that investigated the mechanisms of aging-related changes of NMJ; (3) full-text literature published in English;

Exclusion criteria were: (1) non-English-language papers; (2) without full-text access; (3) not NMJ AChRs-related; (4) NMJ pathological disease-related; (5) without comparisons among different ages; (6) review articles; (7) reports published as conference abstracts.

### Selection of Studies

Study selection was conducted by two independent reviewers. First screening excluded obviously irrelevant papers based on titles and abstracts. The remaining potentially relevant articles were reviewed according to the inclusion and exclusion criteria. Disagreements were resolved by discussion and consensus.

### Data Extraction

The following information was extracted by reviewers: methodology, species, muscle types, age ranges, morphological results, functional results and gene and protein expression data and all the related data on AChRs changes during aging.

### Data Analysis

As the included studies contained only 3 clinical studies and there was high variability in terms of species and methodology, this is not appropriate to conduct a meta-analysis. Hence, a qualitative review was conducted only.

## Results and Discussion

### Results of the Search

**Figure 1:**
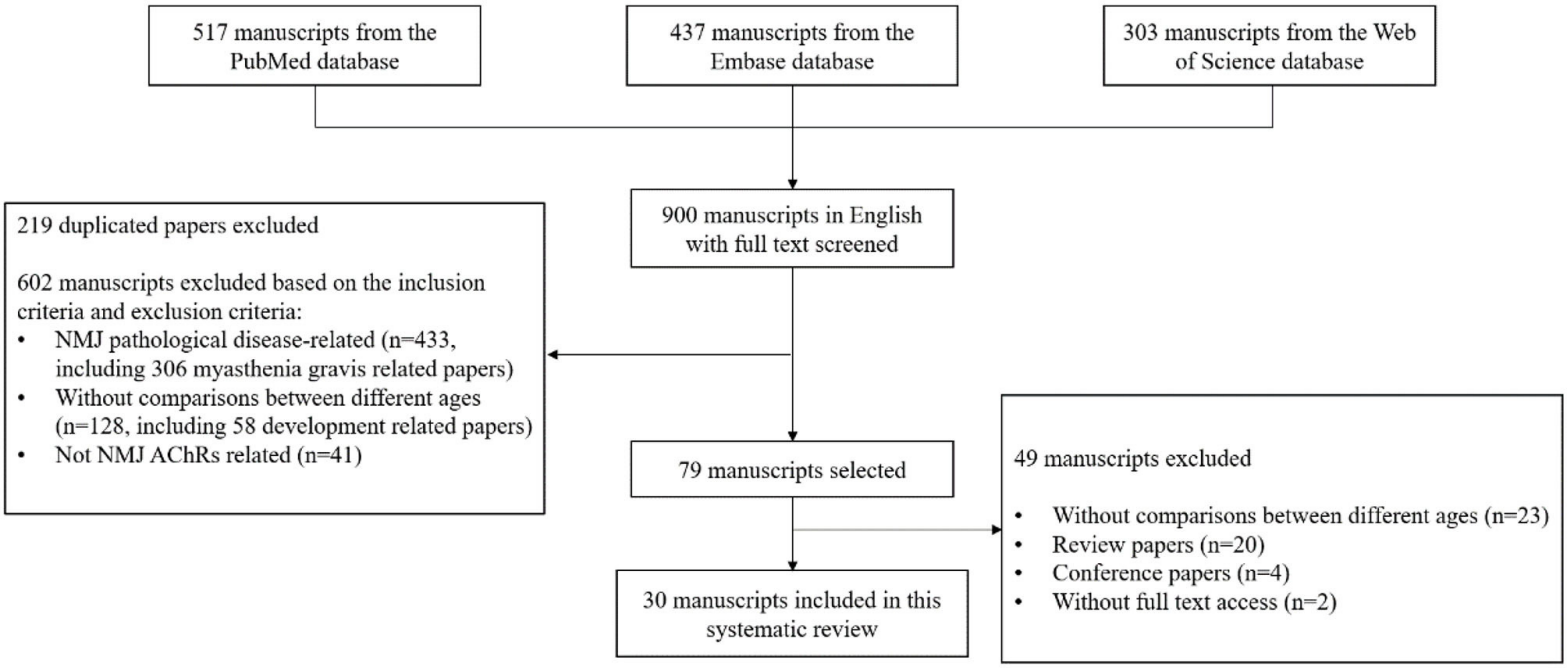
Flow chart showing the selection process of literature search.

After the initial search, the total number of papers was 517 from PubMed, 437 from Embase and 303 from Web of Science databases. 900 papers in English with full text were sorted out for further selection. After reviewing titles and abstracts of the 900 papers, 219 duplicated papers were excluded, 602 papers were excluded based on the selection criteria, and 79 of them were identified as potentially relevant for further examination. After further screening of remaining articles, 49 were excluded, in which 23 had no comparisons among different ages, 20 were review papers 4 were conference reports and 2 had no full text access. Therefore, 30 manuscripts were included for the systematic review (Courtney and Steinbach, 1981; Banker et al., 1983; Oda, 1984; Herscovich and Gershon, 1987; Smith and Chapman, 1987; Anis and Robbins, 1988; Smith et al., 1990; Prakash and Sieck, 1998; Connor et al., 2002; Deschenes and Wilson, 2003; Apel et al., 2009; Mcmullen and Andrade, 2009; Suzuki et al., 2009; Kulakowski et al., 2011; Li et al., 2011; Samuel et al., 2012; Cheng et al., 2013; Deschenes et al., 2013; Personius and Parker, 2013; Aare et al., 2016; Willadt et al., 2016; Xie et al., 2016, 2018; Hughes et al., 2017; Liu et al., 2017; Snyder-Warwick et al., 2018; Zhao et al., 2018; Soendenbroe et al., 2019, 2020; Uchitomi et al., 2019). The flow chart presenting the selection process is shown in Figure 1.

### Characteristics of the Included Studies

The 30 selected studies were conducted from 1981 to 2020: 14 rat studies (Courtney and Steinbach, 1981; Smith and Chapman, 1987; Smith et al., 1990; Prakash and Sieck, 1998; Connor et al., 2002; Deschenes and Wilson, 2003; Apel et al., 2009; Mcmullen and Andrade, 2009; Suzuki et al., 2009; Deschenes et al., 2013; Aare et al., 2016; Xie et al., 2016, 2018; Hughes et al., 2017), 13 mouse studies (Banker et al., 1983; Herscovich and Gershon, 1987; Anis and Robbins, 1988; Kulakowski et al., 2011; Li et al., 2011; Samuel et al., 2012; Cheng et al., 2013; Personius and Parker, 2013; Willadt et al., 2016; Liu et al., 2017; Snyder-Warwick et al., 2018; Zhao et al., 2018; Uchitomi et al., 2019) and 3 human studies (Oda, 1984; Soendenbroe et al., 2019, 2020). The characteristics of the included studies are shown in Table 1.

**Table 1:** Characteristics of included studies.

**Study**	**Strain, species**	**Age range**	**Investigated muscle group(s)**	**Method(s) employed for evaluation**
Courtney and Steinbach (1981)	Lewis rat	100–917 days	Diaphragm and sternomastoid muscle	Radioassay with ^125^I conjugated α-BuTx
Banker et al. (1983)	CBF-1 mouse	8–12 and 29–35 months	EDL, soleus, gluteus maximus, diaphragm, and EDC	Radioassay with ^125^I conjugated α-BuTx; Electron microscopy Electrophysiology Mechanical test
Oda (1984)	Human	32–76 years	Intercostal muscle	Radioassay with ^125^I conjugated α-BuTx
Herscovich and Gershon (1987)	C57BL/6J mouse	6 and 28 months	Gastrocnemius, quadriceps, and diaphragm	Radioassay with ^125^I conjugated α-BuTx
Smith and Chapman (1987)	Fisher 344 rat	10 and 28 months	Diaphragm	Radioassay with ^125^I conjugated α-BuTx Immunofluorescence with fluorescein-conjugated α-BuTx
Anis and Robbins (1988)	C57BL mouse	7–8 and 24–25 months	Soleus	Radioassay with ^125^I conjugated α-BuTx
Smith et al. (1990)	Fisher 344 rat	10 and 25–28 months	Diaphragm	Radioassay with ^125^I conjugated α-BuTx Immunofluorescence with fluorescein-conjugated α-BuTx Electrophysiology
Prakash and Sieck (1998)	Fisher 344 rat	6 and 24 months	Diaphragm	Immunofluorescence with fluorescein-conjugated α-BuTx
Connor et al. (2002)	F344*BN F1 rat	9 and 24 months	Thyroarytenoid muscle	Immunofluorescence with fluorescein-conjugated α-BuTx
Deschenes and Wilson (2003)	Fisher 344 rat	8 and 22 months	Soleus	Immunofluorescence with fluorescein-conjugated α-BuTx
Apel et al. (2009)	F344*BN F1 rat	4 and 24 months	Soleus, gastrocnemius	Immunofluorescence with fluorescein-conjugated α-BuTx
Mcmullen and Andrade (2009)	F344*BN F1 rat	6, 18, and 30 months	Thyroarytenoid and PCA	Immunofluorescence with fluorescein-conjugated α-BuTx Electron microscopy Mechanical test
Suzuki et al. (2009)	Wistar rats	2, 10, 24, and 30 months	Diaphragm	Immunofluorescence with fluorescein-conjugated α-BuTx
Kulakowski et al. (2011)	C57BL/6 mouse	6 and 24 months	Soleus	Immunofluorescence with fluorescein-conjugated α-BuTx Mechanical test
Li et al. (2011)	C57BL/6 mouse	3 weeks−26 months	Sternomastoid muscle	Immunofluorescence with fluorescein-conjugated α-BuTx
Samuel et al. (2012)	C57BL/6 mouse	3–4 and 24–28 months	Triangularis sterni muscle and diaphragm	Immunofluorescence with fluorescein-conjugated α-BuTx
Cheng et al. (2013)	C57BL/6J mouse	2, 14, 19, 22, 25, and 28 months	TA	Immunofluorescence with fluorescein-conjugated α-BuTx
Deschenes et al. (2013)	Fisher 344 rat	8 and 24 months	Soleus, EDL	Immunofluorescence with fluorescein-conjugated α-BuTx
Personius and Parker (2013)	C57BL/6J mouse	3, 12, and 24 months	Soleus	Immunofluorescence with fluorescein-conjugated α-BuTx
Aare et al. (2016)	Fisher 344 rat	8 and 35–36 months	Vastus lateralis	Immunofluorescence with fluorescein-conjugated α-BuTx
Willadt et al. (2016)	C57BL/6J mouse	12–14 and 26–28 months	Diaphragm and EDL	Immunofluorescence with fluorescein-conjugated α-, γ- nAChRs antibody Electrophysiology
Xie et al. (2016)	Sprague-Dawley rat	5 and 20 months	TA and diaphragm	Immunofluorescence with fluorescein-conjugated α-, γ- nAChRs antibody Mechanical test
Hughes et al. (2017)	Fisher 344 rat	9 and 29 months	EDL, gastrocnemius, plantaris, TA, vastus lateralis, and semimembranosus	—
Liu et al. (2017)	C57BL/6J mouse	3, 6, 12, 18, and 24 months	TA, diaphragm, and EDL	Immunofluorescence with fluorescein-conjugated α-BuTx
Snyder-Warwick et al. (2018)	C57BL/6J mouse	3–33 months	Sternomastoid muscle	Immunofluorescence with fluorescein-conjugated α-BuTx
Xie et al. (2018)	Sprague-Dawley rat	4 and 16 months	TA	Electrophysiology
Zhao et al. (2018)	C57BL/6 mouse	3 and 24 months	TA, diaphragm, and gastrocnemius	Immunofluorescence with fluorescein-conjugated α-BuTx Electrophysiology Mechanical test
Soendenbroe et al. (2019)	Human	65–94 years	Vastus lateralis	—
Uchitomi et al. (2019)	C57BL/6J mouse	8 weeks, 4 and 28 months	Gastrocnemius, TA, and EDL	—
Soendenbroe et al. (2020)	Human	20–90 years	Vastus lateralis	—

*EDL, extensor digitorum longus; EDC, extensor digitorum communis; PCA, posterior cricoarytenoid; TA, tibialis anterior; α-BuTx, α-bungarotoxin*.

Among the 14 rat studies, 6 used the diaphragm (Courtney and Steinbach, 1981; Smith and Chapman, 1987; Smith et al., 1990; Prakash and Sieck, 1998; Suzuki et al., 2009; Xie et al., 2016), 2 used laryngeal muscles (Connor et al., 2002; Mcmullen and Andrade, 2009), 3 used soleus (Deschenes and Wilson, 2003; Apel et al., 2009; Deschenes et al., 2013), 1 used plantaris (Hughes et al., 2017), 2 used gastrocnemius (Apel et al., 2009; Hughes et al., 2017), 3 used tibialis anterior (TA) (Xie et al., 2016, 2018; Hughes et al., 2017), 3 used extensor digitorum longus (EDL) (Deschenes et al., 2013; Hughes et al., 2017; Liu et al., 2017), 2 used vastus lateralis (Aare et al., 2016; Hughes et al., 2017) 1 used semimembranosus (Hughes et al., 2017), and 1 used sternomastoid muscle (Courtney and Steinbach, 1981). Among the 13 mouse studies, 6 used diaphragm (Banker et al., 1983; Herscovich and Gershon, 1987; Samuel et al., 2012; Willadt et al., 2016; Liu et al., 2017; Zhao et al., 2018), 4 used soleus (Banker et al., 1983; Anis and Robbins, 1988; Kulakowski et al., 2011; Personius and Parker, 2013), 3 used gastrocnemius (Herscovich and Gershon, 1987; Zhao et al., 2018; Uchitomi et al., 2019), 4 used TA (Cheng et al., 2013; Liu et al., 2017; Zhao et al., 2018; Uchitomi et al., 2019), 3 used EDL (Banker et al., 1983; Willadt et al., 2016; Uchitomi et al., 2019), 1 used gluteus maximus (Banker et al., 1983), 1 used extensor digitorum communis (EDC) (Banker et al., 1983), 1 used quadriceps (Herscovich and Gershon, 1987), 2 used sternomastoid muscle (Li et al., 2011; Snyder-Warwick et al., 2018) and 1 used triangularis sterni (Samuel et al., 2012). The 3 human studies used intercostal muscles and vastus lateralis (Oda, 1984; Soendenbroe et al., 2019, 2020).

Among the 14 rat studies, 2 used rats from 2–3 to 30 months old (Courtney and Steinbach, 1981; Suzuki et al., 2009), 3 used 9–10 and 28–29 months old rats (Smith and Chapman, 1987; Smith et al., 1990; Hughes et al., 2017), 4 used 24 months old rats as the old group and compared with 4, 6, 8, and 9 months old rats, respectively (Prakash and Sieck, 1998; Connor et al., 2002; Apel et al., 2009; Deschenes et al., 2013), 1 used 6, 18, and 30 months old rats (Mcmullen and Andrade, 2009), 1 used 5 and 20 months old rats (Xie et al., 2016), 1 used 8 and 22 months old rats (Deschenes and Wilson, 2003), 1 used 8 and 35–36 months old rats (Aare et al., 2016), 1 used 4 and 16 months old rats (Xie et al., 2018). Among the 13 mouse studies, 1 used 8–12 and 29–35 months old mice (Banker et al., 1983), 1 used 6 and 28 months old mice (Herscovich and Gershon, 1987), 1 used 7–8 and 24–25 months old mice (Anis and Robbins, 1988), 1 used 12–14 and 26–28 months old mice (Willadt et al., 2016), 2 used 24 months old mice as the old group and compared with 3 and 6 months old mice (Kulakowski et al., 2011; Zhao et al., 2018), 1 used mice from 3 weeks to 26 months old (Li et al., 2011), 1 used 3–4 and 24–28 months old mice (Samuel et al., 2012), 1 used mice from 2 to 28 months old (Cheng et al., 2013), 1 used 3, 12, and 24 months old mice (Personius and Parker, 2013), 1 used 3, 6, 12, 18, and 24 months old mice (Liu et al., 2017), 1 used 8 weeks, 4 and 28 months old mice (Uchitomi et al., 2019), 1 used mice from 3 to 33 months old (Snyder-Warwick et al., 2018). The age range of 3 human studies was 32–76 years, 65–94 years, and 20–90 years old (Oda, 1984; Soendenbroe et al., 2019, 2020).

### Aging-Associated Changes in NMJ Morphology and Function

#### Morphological Changes in NMJ

##### Radioassay With α-Bungarotoxin (α-BuTx) Conjugated With ^125^I

Two kinds of AChRs identified at NMJ were classified as “junctional” and “extra-junctional” depending on their location relative to nerve terminals (Smith et al., 1990). The number of either junctional or extra-junctional AChRs would change during aging.

*Junctional AChRs Number* EDL and sternomastoid muscle were typical fast-twitch muscles (Augusto et al., 2004; Giuriati et al., 2018), while junctional AChRs number of the two muscles decreased with aging in both rats and mice (Courtney and Steinbach, 1981; Banker et al., 1983). But this reduction was not observed in mouse gastrocnemius which was also a fast-twitch muscle (Banker et al., 1983; Augusto et al., 2004). Besides, there was no change of junctional AChRs number in mouse soleus that is a well-known slow-twitch muscle (Banker et al., 1983; Anis and Robbins, 1988). Although diaphragm had a larger proportion of fast myosin isoforms, MHC IIa accounted for a larger proportion in diaphragm (Louboutin et al., 1993; Greising et al., 2015), which was different from EDL and sternomastoid muscle with more MHC IIb (Augusto et al., 2004; Giuriati et al., 2018). Banker *et al*. and Herscovich *et al*. reported no changes of junctional AChRs number in mouse diaphragm during aging (Banker et al., 1983; Herscovich and Gershon, 1987). In contrast, Smith *et al*. found junctional AChRs number would increase by more than 80% during aging in diaphragm between 10 months old and 28 months old rats (Smith and Chapman, 1987; Smith et al., 1990). Hence, the different changes of AChRs number in diaphragm among the four studies may be explained by the different animals used (mice in Banker *et al*. and Herscovich *et al*.; rats in Smith *et al*.).

To investigate the specific binding kinetics between α-BuTx and junctional AChRs, Smith *et al*. reported association rate constant between ^125^I-α-BuTx and junctional AChRs decreased by 61% during aging, while the proportion of low-affinity sites in diaphragm junctional AChRs increased from 50.2% in 10 months to 74.6% in 28 months (Smith and Chapman, 1987; Smith et al., 1990).

*Extra-Junctional AChRs Number* Both Courtney *et al*. and Smith *et al*. found significantly higher extra-junctional AChRs during aging in rat sternomastoid muscle fast fibers (Courtney and Steinbach, 1981) and rat diaphragm (Smith and Chapman, 1987; Smith et al., 1990). In contrast, another study found no muscle fibers of intercostal muscle contained the extra-junctional AChRs from 32 to 76 years old autopsied cases (Oda, 1984).

##### Immunofluorescence With Fluorescein-Conjugated α-BuTx

Besides the number, endplate AChRs also experienced morphological changes during aging: from plaque-like appearance in developing period to pretzel-like morphology in adulthood. With increasing age, the continuous junctional folds of AChRs would become fragmented into a series of islands. AChRs morphology could be assessed in three different aspects: endplate size, endplate fragmentation and overlap between nerve terminals and AChRs.

*Endplate Size* The changes of endplate size during aging were quite variable in different studies. AChRs area/perimeter and endplate area/perimeter are the four most common parameters assessing endplate size in immunofluorescence.

Most studies reported a decline of AChRs area in old animals, such as rat thyroarytenoid muscle (6–30 months old) and mouse TA (14 and 19 months old) (Connor et al., 2002; Mcmullen and Andrade, 2009; Cheng et al., 2013). Thyroarytenoid muscle was mainly composed of MHC IIb, MHC IIx and MHC eo (a special isoform of MHCs in the thyroarytenoid muscle), while TA had a higher proportion of MHC IIb (Augusto et al., 2004; Rhee et al., 2004). EDL was also mainly composed of MHC IIb, but there was no significant difference of AChRs and endplate area during aging in both mice (12–14 and 26–28 months old) and rats (8 and 24 months old) (Deschenes et al., 2013; Willadt et al., 2016). Changes of AChRs area in soleus were inconsistent among different studies. Kulakowski *et al*. and Personius *et al*. found AChRs area were expanded in old mice (3–24 months old) (Kulakowski et al., 2011; Personius and Parker, 2013), but AChRs area in rats were similar among 4, 8, and 24 months old (Apel et al., 2009; Deschenes et al., 2013). In another study, Deschenes *et al*. reported reduced AChRs and endplate area in rats of 22 vs. 8 months (Deschenes and Wilson, 2003). Considering diaphragm composed of MHC I, MHC IIa, MHC IIb and MHC IIx, Prakash *et al*. and Suzuki *et al*. used diaphragm to investigate myofiber type specific AChRs appearance changes with aging in rats and reported that AChRs and endplate area in type IIb and IIx fibers increased from 2 to 30 months old, yet there were no significant differences in type I and IIa fibers (Prakash and Sieck, 1998; Suzuki et al., 2009).

*Endplate Fragmentation* Endplate structure was remarkably stable as pretzel-like appearance throughout most of the life (Lichtman et al., 1987). Nevertheless, this structure would become unstable during aging, for example, aged endplate would fragment into small islands. Although there is still no consensus to define and classify fragmentation, almost all studies concluded that endplate would fragment with increasing age.

In fast-twitch muscles, sternomastoid muscle and TA, the incidence of fragmented AChRs dramatically increased during aging in mice (Li et al., 2011; Cheng et al., 2013; Liu et al., 2017; Snyder-Warwick et al., 2018; Zhao et al., 2018). Li *et al*. reported the incidence of fragmented junctions (with 10 or more fragments of endplate) increased dramatically from <25% at 18 months to >80% at 22 months in sternomastoid muscle (Li et al., 2011). Cheng *et al*. defined fragmented endplates as those with 5 or more AChRs clusters and Liu *et al*. defined fragmented endplate as a patch like area devoid of elaborate branches >5 um in length. They both observed increased fragmented endplate in TA of 22–24 months old (Cheng et al., 2013; Liu et al., 2017). Mouse sternomastoid muscle of 3 and 9 months showed normal pretzel-like endplate at each NMJ, but ~34% endplate showed partial or full fragmentation at 14 months of age and increased drastically between 17 and 33 months. By 33 months of age, only ~12% of endplate presented normal pretzel-like morphology (Snyder-Warwick et al., 2018). The same morphological changes were also observed in TA, which endplate in 3 months appeared as characteristic pretzel-like structures, but a majority (~80%) of endplate were fragmented by 24 months (Zhao et al., 2018). Besides, higher proportion of fragmented endplate in sternomastoid muscle was also observed in rats (Courtney and Steinbach, 1981). Typical slow-twitch muscle soleus also showed more discrete AChRs fragments in old rats (4 and 24 months old) and mice (3–24 months old) (Apel et al., 2009; Kulakowski et al., 2011; Personius and Parker, 2013). Willadt *et al*. and Liu *et al*. reported significantly fragmented AChRs in old diaphragm as well (Willadt et al., 2016; Liu et al., 2017). Although most studies found that endplate fragmented with aging, one study reported that AChRs clusters (3 or more NMJs located within no more than 5 um apart) significantly decreased with aging among 6, 18, and 30 months old in both PCA and thyroarytenoid muscle of rats (Mcmullen and Andrade, 2009). Another study found no difference of fragments in mouse EDL between 12–14 and 26–28 months (Willadt et al., 2016).

*Overlap Between Nerve Terminals and AChRs* Denervation during aging was also reported in many studies and evaluated by the overlap between nerve terminals and AChRs. Changes of this parameter could be explained by area changes of either nerve terminals or AChRs, because it represents the relative relationship between pre- and post-synapse. In this review, we would not discuss morphological changes of pre-synapse but mainly focus on the change of overlap area between nerve terminals and AChRs during aging.

Denervation was widely observed in included mouse studies: slow-twitch soleus, fast-twitch TA and respiratory muscle diaphragm all had greater post- to pre-synaptic ratio in old animals (Kulakowski et al., 2011; Cheng et al., 2013; Liu et al., 2017; Zhao et al., 2018). Kulakowski *et al*. reported slightly reduced overlap between nerve terminals and AChRs in 24 months soleus compared with 6–8 months (Kulakowski et al., 2011); Cheng et al. (2013), Liu et al. (2017), and Zhao et al. (2018) also found denervation appearance in TA. Overlap between nerve terminals and AChRs fell from 55% at 19 months to 34% at 25 months in Cheng et al. (2013). Partially (>5 μm length of an AChRs branch was not covered by nerve terminals) and fully denervated (>90% of AChRs cluster was devoid of nerve terminals) AChRs clusters both significantly increased from 6 to 24 months (Liu et al., 2017). This study also reported increased denervation in diaphragm (Liu et al., 2017). Fully innervated AChRs clusters (80–100% neve-endplate overlap) were reduced by approximately 50% in 24 months as compared with 3 months (Zhao et al., 2018). In rat studies, Deschenes *et al*. found no obvious signs of denervation in old slow-twitch soleus and fast-twitch EDL (Deschenes and Wilson, 2003; Deschenes et al., 2013). Besides, there were no apparent differences in the spatial distribution of AChRs relative to the nerve-terminal from 10 to 28 months diaphragm (Smith et al., 1990). But there was only one study reporting higher post- to pre-synaptic ratio in old thyroarytenoid muscle (Connor et al., 2002). Interestingly, Snyder-Warwick *et al*. observed in mouse sternomastoid muscle that AChRs denervation significantly increased from 20% at 14 months to 35% at 17 months, as compared with 3 months old, but decreased again until 33 months (Snyder-Warwick et al., 2018). This may be the reason why Li *et al*. reported no signs of denervation at 22–26 months in mouse sternomastoid muscle (Li et al., 2011).

##### Electron Microscopy

*Ultrastructure of Endplate* One study found that old mouse EDL (30 and 34 months old) showed focal atrophy of post-synaptic folds and nerve terminal retraction from the synaptic folds (Banker et al., 1983). Two studies reported that the proportion of AChRs unoccupied by axon terminals was increased in mouse EDL and rat laryngeal muscle 1(Banker et al., 1983; Mcmullen and Andrade, 2009).

#### Functional Changes in NMJ

It is hard to estimate AChRs function separately and precisely, because the efficient functioning of NMJ relies on the cooperation among different elements, while AChRs is only one part of NMJ. When an action potential reaches nerve terminal, voltage-dependent calcium channels will open and allow calcium to enter the motor neuron. ACh is then delivered from nerve terminals and activates post-synaptic AChRs to produce an action potential, which in turn triggers the opening of voltage-gated dihydropyridine receptors (DHPRs) and ryanodine receptors (RyRs). Eventually, calcium is released from the sarcoplasmic reticulum, leading to force production (Gonzalez-Freire et al., 2014). In this part, AChRs function will be summarized based on two different testing methods: electrophysiology and mechanical test.

##### Electrophysiology

*Miniature Endplate Potential (MEPP)* MEPP is local depolarizing potential elicited by spontaneously released ACh. Reduced MEPP amplitude may indicate fewer AChRs or weaker binding between ACh and AChRs to certain extent (Rocha et al., 2013). MEPP decay time represents the open time of AChRs and prolonged decay time suggests the reduced acetylcholinesterase (AChE) activity or increased binding between ACh and AChRs (Rocha et al., 2013).

One mouse study found that MEPP amplitude only increased with aging in the diaphragm (mainly composed of MHC IIa) (Louboutin et al., 1993; Greising et al., 2015), but there was no significant difference in either typical slow-twitch soleus or typical fast-twitch EDL (8–12 and 29–35 months old). Some parameters related to MEPP amplitude were also measured, but muscle fiber diameter, input resistance and membrane capacitance were found not associated with the increase of MEPP amplitude (Banker et al., 1983). In contrast, one study reported that MEPP amplitude was reduced in left hemi-diaphragm of 24-month mice compared with 3-month mice (Zhao et al., 2018). And another study reported no changes of MEPP amplitude in mouse diaphragm and EDL between 12–14 and 26–28 months old (Willadt et al., 2016).

To further investigate the mechanism of MEPP changes, one study applied AChE inhibition test in rat diaphragm (Smith et al., 1990). Before AChE inhibition, MEPP was similar between 10 and 25–28 months (amplitudes, time to peak and decay time). After AChE inhibition, the magnitudes of the increases in both amplitude and decay time were significantly greater in old diaphragm. Following AChE inhibition, MEPP decay time was governed primarily by the diffusion rate of ACh away from the AChRs, so old AChRs may have increased availability to bind ACh.

*Evoked Endplate Potentials (EPP)* Unlike MEPP, EPP is triggered by action potential reaching the motor neuron terminals, ACh is then released and binds to AChRs, leading to post-synaptic membrane depolarization. Hence, EPP can be considered as comprising multiple MEPPs.

Banker *et al*. recorded EPP in the standard saline containing 2–6 uM (+)-tubocurarine chloride, so larger EPP amplitude in curarized muscles indicated diminished AChRs' curare sensitivity or stronger binding between ACh and AChRs (Banker et al., 1983). In both fast-twitch EDL and slow-twitch soleus of old mice (34–35 months old), there was a pronounced increase in the EPP amplitude at both 2 Hz and 20 Hz. This outcome combined with the absence of MEPP amplitude changes in the same study, revealing that the number of released ACh quanta was increased at the stimulation frequency of 2 and 20 Hz. Another study calculated released ACh quanta by the ratio of the mean peak amplitude of the evoked endplate current (EPC) to that of the miniature endplate current (MEPC) and found that amplitude of EPC at 1 Hz was significantly larger in diaphragm exposed to 10 nM α -BuTx of 26–28 months compared with 12–14 months but there was no significant difference of ACh quanta between two ages (Willadt et al., 2016).

*Compound Muscle Action Potentials (CMAP)* Different from EPP which was recorded intracellularly at endplate regions of muscle fibers (Banker et al., 1983), CMAP was recorded by electrode inserted directly into the target muscle and considered as a summation of all individual muscle fiber action potentials (Zhao et al., 2018). With stimulation frequency at 20 and 40 Hz in sciatic nerve, CMAP amplitude in left gastrocnemius (mainly composed of MHC IIb) (Augusto et al., 2004), was smaller in 24 months at 10th stimuli compared with 3 months in mice. At 40 Hz, CMAP amplitude showed significant reduction as early as 4th stimuli in 24 months mice (Zhao et al., 2018). In another fast-twitch muscle TA (also mainly composed of MHC IIb) (Augusto et al., 2004), CMAP amplitude was significantly reduced in 16 months old rats with stimulation at 1 Hz in sciatic nerve compared with 4 months (Xie et al., 2018). But in diaphragm (mainly composed of MHC IIa) (Louboutin et al., 1993; Greising et al., 2015), there were no significant changes in CMAP amplitude at 5 Hz in mice between 12–14 and 26–28 months old, then MHC composition may have effects on CMAP amplitude (Willadt et al., 2016).

##### Mechanical Test

*Motor Unit Function* NMJ is a precise transmission device connecting motor neuron to skeletal muscle. Measuring muscle contractile function by stimulating motor neuron can evaluate the overall function of motor unit. Comparing muscle contractile differences triggered by nerve and muscle stimulus will provide a different perspective to investigate NMJ function, which also reflects the function of AChRs.

A recent study found that twitch and tetanic force of hindlimb stimulated by sciatic nerve decreased in 24 months compared with 3 months in mice (Zhao et al., 2018). To investigate the detailed mechanisms, two other studies used different methods to block NMJ and revealed the characteristics of NMJ function during aging. One study measured indirect twitch force of hindlimb muscles by stimulating nerve under conditions of reduced calcium-to-magnesium ratio. Transmitter release was reduced, whereas the direct twitch was unaffected under this condition. Therefore, reduced amplitude of the indirect twitch gave a relative measure of synaptic efficiency. Young fast-twitch EDL and slow-twitch soleus both showed significantly greater depression of twitch amplitude than old muscles of 28–31 months mice. However, in the same study, young and old diaphragm showed no difference in twitch reduction (Banker et al., 1983).

Another study used tubocurarine to block Ach-AChRs binding and found that force produced by nerve stimulation at any tubocurarine concentration decreased significantly with aging in rat laryngeal muscle, which indicated greater tubocurarine sensitivity and functional denervation during aging (Mcmullen and Andrade, 2009). In comparing NS/MS (the ratio of force in response to nerve and muscle stimulation) at different tubocurarine concentrations, the ratio of PCA which was mainly composed of MHC IIx (Rhee et al., 2004), remained >0–5 uM tubocurarine, and it was 0 at 10 and 20 uM in 6 months rats. In contrast, the decrease in NS/MS in PCA of 30 months rats was significantly greater than at 6 months starting from the lowest concentration used (0.5 uM), and NS/MS became <10% at 1 uM and about 0 at 2 uM tubocurarine. But the response to tubocurarine was similar in the thyroarytenoid muscle (mainly composed of MHC IIb, x and eo) between young and old muscles (Rhee et al., 2004; Mcmullen and Andrade, 2009). Recently, more NMJ studies used neurotransmission failure (NF = (F-MF) / (1-MF), where F was percent force loss during nerve stimulation and MF was percent force loss in direct muscle stimulation) to evaluate NMJ function. One study found that NF was significantly increased in 24 months soleus compared with 6–8 months in mice (Kulakowski et al., 2011).

### Discussion on NMJ Morphological and Functional Changes

As the interface between nerve and skeletal muscle, NMJ ensures effective transmission from chemical signals in nerve terminals to electrical signals in skeletal muscles. Since this stimulation is essential for normal muscle contraction and muscle mass maintenance, NMJ structure and function impairment is suggested to play a pivotal role in sarcopenia-related decline of muscle mass and muscle power generation (Faulkner et al., 2007; Casati et al., 2019). To achieve the maximal transmission efficiency, pre- and post-synaptic membranes exhibit the same branch pattern, termed as “pretzel-like” (Rudolf et al., 2014). Rowan *et al*. reported denervation in senescent skeletal muscles and concluded this was the major contribution to muscle fiber atrophy with aging (Rowan et al., 2012). To adapt to the degeneration of pre-synaptic nerve terminals or influence of the muscle fiber atrophy underlying synapse with increasing age, post-synaptic AChRs undergoes structural reorganization, referred to as “fragmentation” (Rudolf et al., 2014). Deschenes *et al*. also found morphological remodeling of AChRs preceded sarcopenia-related alterations in skeletal muscles during aging, including increased AChRs area and perimeter (Deschenes et al., 2010). Investigating morphological and functional alterations of AChRs may help to understand the detailed mechanisms during the pathogenesis of sarcopenia and provide new directions of prediction and therapeutic targets for this syndrome.

Different muscle groups in different species presented different changes in AChRs during aging. Skeletal muscles can be classified into slow-twitch and fast-twitch muscles in terms of the proportions of myosin heavy chain (MHC) isoforms, namely the slow-twitching MHC I and three fast-twitching MHC IIa, IIb, and IIx (Augusto et al., 2004). Differences in the changes of AChRs morphology and function in relations to various muscle fiber types and species and MHC compositions in different muscle groups are summarized in Table 2. Notably, sarcopenia is also accompanied with obviously smaller type II fibers and commonly unaffected type I fibers (Miljkovic et al., 2015).

**Table 2:** NMJ morphology and function differences in various muscles and species with different MHC composition.

**Muscle type**	**Major MHC type**	**Species**	**Morphological changes during aging**	**Functional changes during aging**
**MOSTLY SLOW-TWITCHING**				
Soleus	MHC I, IIa (Augusto et al., 2004)	Mouse	No changes of junctional AChRs number (Banker et al., 1983; Anis and Robbins, 1988) AChRs area and perimeter were expanded (Kulakowski et al., 2011; Personius and Parker, 2013) Increased fragmentation (Kulakowski et al., 2011; Personius and Parker, 2013) Increased signs of denervation (Kulakowski et al., 2011)	No changes of MEPP amplitude (Banker et al., 1983) Increased EPP amplitude (Banker et al., 1983) Smaller twitch amplitude depression after nerve stimulation in low calcium-magnesium ratio condition (Banker et al., 1983) Increased neurotransmission failure (Kulakowski et al., 2011)
		Rat	No changes of AChRs area (Apel et al., 2009; Deschenes et al., 2013) AChRs perimeter was expanded (Deschenes et al., 2013) Reduced AChRs and endplate area (Deschenes and Wilson, 2003) Increased fragmentation (Apel et al., 2009) No signs of denervation (Deschenes and Wilson, 2003; Deschenes et al., 2013)	—
**MOSTLY FAST-TWITCHING**				
Diaphragm	MHC IIa (Louboutin et al., 1993; Greising et al., 2015)	Mouse	No changes of junctional AChRs number (Banker et al., 1983; Herscovich and Gershon, 1987) No changes of AChRs area (Willadt et al., 2016) Increased fragmentation (Willadt et al., 2016; Liu et al., 2017) Increased denervation (Liu et al., 2017)	Increased MEPP amplitude (Banker et al., 1983) Reduced MEPP amplitude (Zhao et al., 2018) No changes of MEPP amplitude (Willadt et al., 2016) No changes of CMAP amplitude decline (Willadt et al., 2016) No twitch amplitude differences after nerve stimulation in low calcium-magnesium ratio condition (Banker et al., 1983)
		Rat	Increased junctional and extra-junctional AChRs number (Smith and Chapman, 1987; Smith et al., 1990) Expanded AChRs and endplate area in type IIx and IIb fibers, but no changes of AChRs area in type I and IIa fibers (Prakash and Sieck, 1998; Suzuki et al., 2009) Increased AChR-γ and AChR-α7 density (Xie et al., 2016) No signs of denervation (Smith et al., 1990)	No changes of MEPP amplitude (Smith et al., 1990) Greater magnitude of MEPP amplitude increase after AChE inhibition (Smith et al., 1990)
Sternomastoid muscle	MHC IIa, IIb (Giuriati et al., 2018)	Mouse	Increased fragmentation (Li et al., 2011; Snyder-Warwick et al., 2018) No signs of denervation (Li et al., 2011)	—
		Rat	Decreased junctional AChRs number (Courtney and Steinbach, 1981) Increased fragmentation (Courtney and Steinbach, 1981)	—
EDL	MHC IIb (Augusto et al., 2004)	Mouse	Decreased junctional AChRs number (Banker et al., 1983) No changes of AChRs area (Willadt et al., 2016) No changes of fragmentation (Willadt et al., 2016)	Increased EPP amplitude (Banker et al., 1983) No changes of MEPP amplitude (Banker et al., 1983; Willadt et al., 2016) Smaller twitch amplitude depression after nerve stimulation in low calcium-magnesium ratio condition (Banker et al., 1983)
		Rat	No changes of AChRs and endplate area (Deschenes et al., 2013) No signs of denervation (Deschenes et al., 2013) No signs of dispersion (Deschenes et al., 2013)	—
Gastrocnemius	MHC IIb (Augusto et al., 2004)	Mouse	No changes of junctional AChRs number (Herscovich and Gershon, 1987)	Easier to appear CMAP amplitude reduction (Zhao et al., 2018)
		Rat	—	—
TA	MHC IIb (Augusto et al., 2004)	Mouse	Declined AChRs area (Cheng et al., 2013) Increased fragmentation (Cheng et al., 2013; Liu et al., 2017; Zhao et al., 2018) Increased denervation (Cheng et al., 2013; Liu et al., 2017; Zhao et al., 2018)	—
		Rat	Increased γ-nAChR and α7-nAChR density (Xie et al., 2016)	Reduced CMAP amplitude, increased CMAP duration, nerve conduction velocity and latency period (Xie et al., 2018)
PCA	MHC IIx (Rhee et al., 2004)	Mouse	—	—
		Rat	Decreased fragmentation (Mcmullen and Andrade, 2009)	Increased response to tubocurarine (Mcmullen and Andrade, 2009)
Thyroarytenoid muscle	MHC IIb, IIx, eo (Rhee et al., 2004)	Mouse	—	—
		Rat	Declined AChRs area (Connor et al., 2002) Decreased fragmentation (Mcmullen and Andrade, 2009) Increased denervation (Connor et al., 2002)	No differences of response to tubocurarine (Mcmullen and Andrade, 2009)

*EDL, extensor digitorum longus; TA, tibialis anterior; PCA, posterior cricoarytenoid; MHC, myosin heavy chain; AChRs, acetylcholine receptors; EPP, evoked end-plate potential; MEPP, miniature end-plate potential; CMAP, compound muscle action potential*.

Endplate fragmentation and reduced overlapping between nerve terminal and AChRs are commonly observed and widely reported in either slow or fast-twitch muscles of old animals (Apel et al., 2009; Kulakowski et al., 2011; Li et al., 2011; Cheng et al., 2013; Personius and Parker, 2013; Willadt et al., 2016; Liu et al., 2017; Snyder-Warwick et al., 2018; Zhao et al., 2018), which can explain reduced CMAP amplitude with aging that can be considered as an overall function of all individual NMJs (Xie et al., 2018). Therefore, increased endplate fragmentation and denervation may contribute to overall decreased efficiency or function of NMJ.

AChRs area is another important parameter to assess morphological changes of endplate in immunofluorescence staining with fluorescein-conjugated α-BuTx. However, changes in AChRs area could not be sufficiently explained or generalized by muscle type and species only. Thus, progression of muscle atrophy needs to be taken into account. Although TA and EDL were both mainly composed of MHC IIb, muscle atrophy was commonly observed in TA (Liu et al., 2017; Zhao et al., 2018; Uchitomi et al., 2019), while muscle fiber cross-sectional area had no significant changes with aging in EDL (Banker et al., 1983; Willadt et al., 2016). This could explain declined AChRs area in old TA (Cheng et al., 2013), while AChRs area was similar between young and old EDL (Deschenes et al., 2013; Willadt et al., 2016). Cross sectional area was similar in mouse soleus between 24 months and 6–8 months old (Kulakowski et al., 2011), but soleus in rats of 24 months old showed significant atrophy (Deschenes et al., 2013). Therefore, the slow-twitch muscles with higher proportions of MHC I fibers demonstrate enlarging AChRs areas, which could explain expanded AChRs area in 24 months old mouse soleus while rat soleus in 24 months had similar or reduced AChRs area compared with young adult rats (Deschenes and Wilson, 2003; Apel et al., 2009; Kulakowski et al., 2011; Deschenes et al., 2013; Personius and Parker, 2013). Therefore, muscle atrophy could significantly reduce AChRs area during aging, while slow-twitch muscles tend to have enlarged AChRs area.

Skeletal muscles mainly composed of MHC IIb, like the EDL and gastrocnemius (Augusto et al., 2004), showed reduced or no changes of AChRs number during aging (Banker et al., 1983; Herscovich and Gershon, 1987), while there was no significant difference of MEPP amplitude between young and old muscles (Banker et al., 1983; Willadt et al., 2016). Thus, the binding between AChRs and ACh has no changes with increasing age in these muscles. Banker *et al*. also reported an increased release of ACh quanta and this can explain the results of electrophysiology test where CMAP amplitude tended to reduce more easily during several stimulus in old animals (Banker et al., 1983; Zhao et al., 2018). The same increased release of ACh was also observed in typical slow-twitch muscle soleus and this explains increased NF in old soleus (Banker et al., 1983; Kulakowski et al., 2011). There were no significant changes of AChRs number and ACh quanta release in diaphragm during aging which was mainly composed of MHC IIa (Banker et al., 1983; Anis and Robbins, 1988; Louboutin et al., 1993; Greising et al., 2015; Willadt et al., 2016), but one study reported old diaphragm showed increased MEPP amplitude, indicating stronger binding between ACh and AChRs in old diaphragm (Banker et al., 1983; Smith et al., 1990). Reduced association rate constant between ^125^I-α-BuTx and AChRs in old diaphragm can also support this point, because α-BuTx could compete with ACh to conjugate with AChRs (Smith and Chapman, 1987; Smith et al., 1990). After the stimulation is completed, the binding between ACh and AChRs is immediately broken down by AChE and Smith *et al*. reported increased AChE activity in old rat diaphragm, promoting the recruitment of AChRs before next stimulation (Smith and Emmerling, 1988). Therefore, different muscles may have different adaption mechanisms to make up for NMJ morphological degradation, including increased ACh releasing, increased AChE activity or increased affinity between ACh and AChRs.

**Figure 2:**
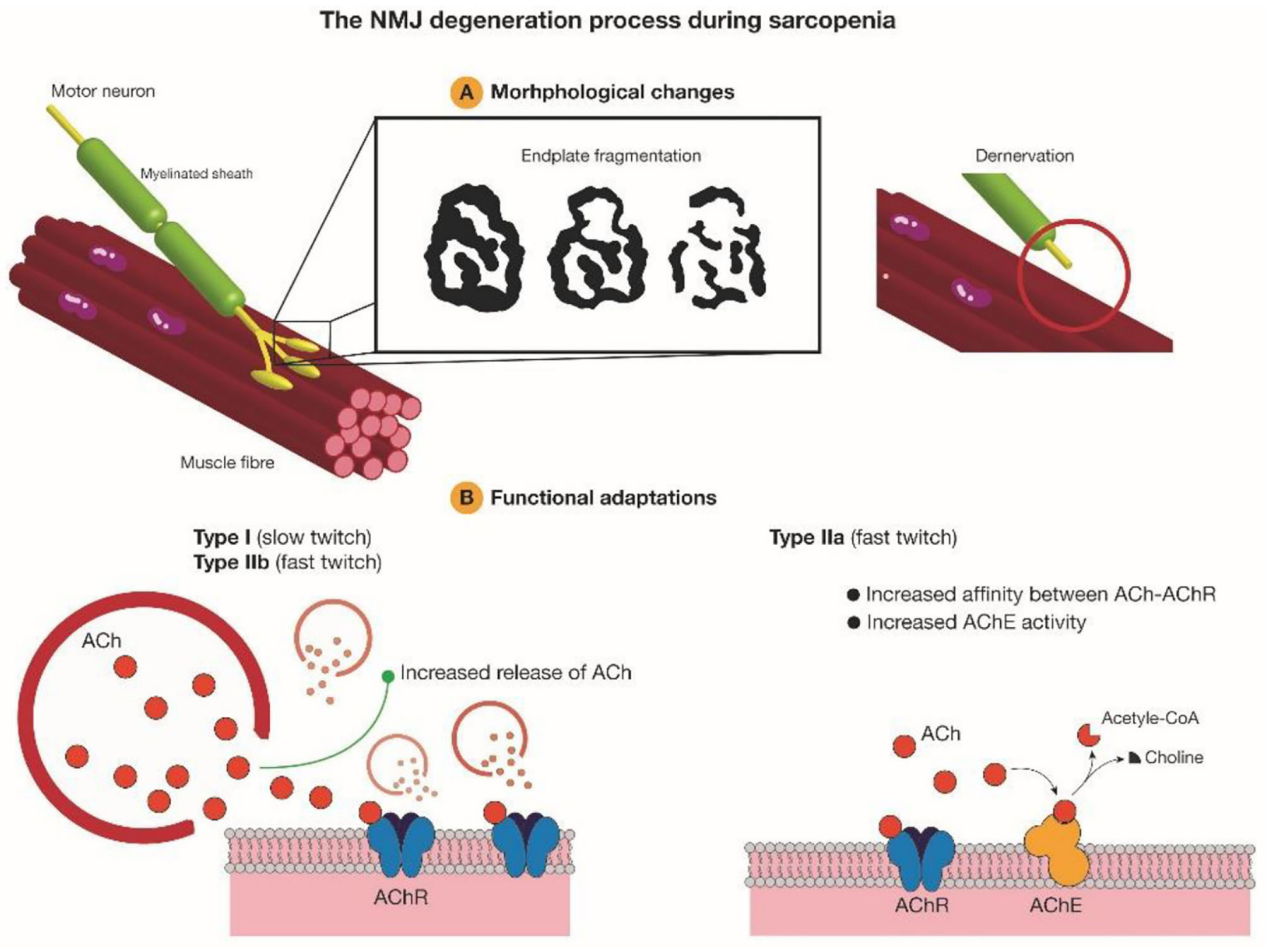
AChRs morphological degeneration and functional adaptations during aging. Increased endplate fragmentation and denervation during aging disrupt precise neurotransmission at NMJ. To compensate and recover normal function, slow-twitch muscles and fast-twitch muscles mainly composed of MHC IIb tend to increase ACh quanta releasing, while skeletal muscles mainly composed of MHC IIa would increase AChE activity and the affinity between AChRs and ACh. Ach, acetylcholine; AChR, acetylcholine receptor; AChE, acetylcholinesterase.

In summary, endplate fragmentation and overlap between nerve terminals and AChRs can be taken as two reliable parameters to assess NMJ degeneration during aging. Tintignac *et al*. and Larsson *et al*. also pointed out muscle atrophy and muscle waste can be triggered by denervation (Tintignac et al., 2015; Larsson et al., 2019), thus these two parameters have potential to be taken as predictors for sarcopenia in the future. Increased endplate fragmentation and decreased overlap between nerve terminals and endplates during aging may disengage the transmission between pre- and post-synapse. Different muscles may have different compensation mechanisms. Slow-twitch muscles and fast-twitch muscles mainly composed of MHC IIb could increase released ACh quanta, while diaphragm mainly composed of MHC IIa could increase the availability of AChRs binding to ACh, but the detailed mechanism requires further investigation (Figure 2).

### Aging-Associated Changes in AChRs-Related Gene Expressions

#### AChRs Subunits

Five types of polypeptide chains, α, β, δ and γ or ε subunit, compose the heteropentamer AChRs in skeletal muscles. These subunits also have some changes of expression level with increasing age. mRNA level of AChR-α and -γ were commonly increased with aging in rats, including gastrocnemius (4 and 24 months old) (Apel et al., 2009), PCA (6 and 30 months old) (Mcmullen and Andrade, 2009), vastus lateralis (8 and 35–36 months old) (Aare et al., 2016), TA and diaphragm (5 and 20 months old) (Xie et al., 2016). In human vastus lateralis, Soendenbroe et al. (2020) also reported increased AChR-α and -γ mRNA expression in elderly women (71–78 years old) compared with young women (20–28 years old), while AChR-β mRNA was decreased with aging (Soendenbroe et al., 2020). However, there was not a consistent result about changes of α subunit mRNA expression during aging in mice: Uchitomi *et al*. found higher mRNA level of AChR-α in mouse skeletal muscles of 28 months (Uchitomi et al., 2019), while Liu *et al*. reported declined mRNA level of α subunit in diaphragm of 24 months old vs. 6 months (Liu et al., 2017). Besides, in a human study, the mRNA level of AChR-γ was reduced from 65 to 94 years old in vastus lateralis, yet this age range was too short and may need more studies to confirm (Soendenbroe et al., 2019). Aging had no significant effects on α and γ subunit protein in rat gastrocnemius which was mainly composed of MHC IIb (Augusto et al., 2004; Apel et al., 2009). PCA (mainly composed of MHC IIx) and thyroarytenoid muscle (mainly composed of MHC IIb, IIx, and eo) were laryngeal muscles, protein level of γ subunit decreased in aged PCA but increased in old thyroarytenoid muscle (Rhee et al., 2004; Mcmullen and Andrade, 2009).

The opposite trend during aging was also reported in the mRNA level of δ subunit between the two laryngeal muscles, increased in old PCA while decreased in old thyroarytenoid muscle between 4 and 24 months old (Mcmullen and Andrade, 2009). Besides, Apel *et al*. found higher mRNA expression of δ subunit in old rat gastrocnemius while Liu *et al*. reported decreased mRNA level of AChR-δ in old mouse diaphragm (Apel et al., 2009; Liu et al., 2017). Interestingly, in another mouse diaphragm study, the protein level of AChR-δ increased with aging between 3 and 24 months old (Zhao et al., 2018).

Both mRNA and protein levels of ε subunit increased in old rat thyroarytenoid muscle (6 and 30 months old) and protein level of this subunit was also higher in old PCA (6 and 30 months old), although mRNA level was similar between young and old PCA (Mcmullen and Andrade, 2009). Another rat study also found higher mRNA level of AChR-ε in old vastus lateralis (8 and 35–36 months old) (Aare et al., 2016). In contrary, mRNA level of ε subunit was reduced in mouse diaphragm of 24 months old compared with 6 months (Liu et al., 2017), while there was no difference during aging at protein level (3 and 24 months old) (Zhao et al., 2018).

#### Myogenic Regulator Factors (MRFs)

NMJ regeneration with increasing age is closely regulated by MRFs, a family of helix-loop-helix transcription factors which include myogenic factors 5 (Myf5), muscle regulatory factor 4 (MRF4), myogenin, and myoblast determination factor (MyoD) (Apel et al., 2009). These factors can form heterodimers with basic Helix-Loop-Helix (bHLH) proteins and bind to E box in the regulatory regions of AChRs (Gilmour et al., 1991; Prody and Merlie, 1991). By regulating the expression of AChRs subunits, MRFs could play a role in AChRs stabilization and destabilization (Ma et al., 2007). Aged rat gastrocnemius showed increased mRNA expression of MRF4 and myogenin. There was no difference in protein expression of myogenin between young and aged groups but MRF4 protein expression was significantly lower in aged animals (Apel et al., 2009).

#### Agrin-LRP4-MuSk-Rapsyn-Dok7

Agrin is a nerve-derived organizer of post-synaptic differentiation in skeletal muscles. Muscle cells also express Agrin, but only motor neurons produce an alternatively spliced isoform called “z-Agrin.” It is bound to the low-density lipoprotein receptor-related protein 4 (LRP4), which triggers the auto-phosphorylation of the muscle-specific kinase (MuSk) and activates MuSk, leading to AChR clustering through receptor-associated protein of the synapse (Rapsyn) (Liu and Chakkalakal, 2018). During this process, docking protein 7 (Dok7) plays an important role as a substrate for MuSk and maintains MuSk tyrosine phosphorylation (Burden et al., 2018). In a word, Agrin-LRP4-MuSk-Rapsyn-Dok7 is necessary for AChRs cluster formation and stabilization.

z-Agrin mRNA level did not differ dramatically between young and old spinal cords in mice. Agrin was precisely opposite to regions of high AChRs density in 3–4 months mouse diaphragm. Some synaptic regions were depleted of Agrin in 24–28 months diaphragm, and immunoreactivity extended into peri-synaptic areas (Samuel et al., 2012). Aare et al. (2016) reported higher level of MuSk mRNA and Rapsyn protein in rat vastus lateralis of 35–36 months vs. 8 months old (Aare et al., 2016). Another study using vastus lateralis also reported higher expression of MuSk mRNA in elderly women (71–78 years old) compared with young women (20–28 years old) (Soendenbroe et al., 2020). But in mouse diaphragm, *Liu et al*. found no significant difference of MuSk mRNA expression between 6 and 24 months old (Liu et al., 2017). Besides, in another mouse diaphragm study, MuSk mRNA was found increased while MuSk phosphorylation was reduced during aging. In the same study LRP4 protein level was reduced by 50% in 24 months synaptic region of diaphragm compared with 3 months, but LRP4 mRNA level was higher in aged mice. Then Zhao *et al*. analyzed the ubiquitination of LRP4 and found the amount of ubiquitinated LRP4 was increased at 24 months compared with 3 months. So LRP4 degradation was increased by proteasome in aged muscles. Protein levels of Agrin, MuSk, Rapsyn, and Dok7 were similar between the young and old samples (Zhao et al., 2018).

#### Dystrophin-Glycoprotein Complex (DGC)

DGC is a transmembrane complex required for AChRs clustering, including three subcomplexes: the cytoplasmic complex comprised of Dystrophin/Utrophin, Dystrobrevin and Syntrophin, the transmembrane Dystroglycan complex and the Sarcoglycan-Sarcospan complex (Gawor and Proszynski, 2018). At the crests of junctional folds, Dystroglycan-β and Dystrophin/Utrophin binds to Rapsyn, associating with the clustering of AChRs (Pilgram et al., 2010). Zhao *et al*. found that Sarcoglycan-α could interact with LRP4 and delay LRP4 degradation, thus stabilizing AChRS clusters (Zhao et al., 2018).

In rat gastrocnemius, the protein levels of Sarcoglycan-α and Syntrophin were higher in 29 vs. 9 months while there were no significant differences of Dystrophin, Dystroglycan-β and Sarcospan (Hughes et al., 2017). In the same study, old TA was shown higher protein expressions of Sarcospan, Sarcoglycan-α and Syntrophin, while Dystroglycan-β protein level showed no significant difference between adult and old group, and Dystrophin protein was reduced in 29 months (Hughes et al., 2017).

#### Tyrosine Kinase B

Full-length tyrosine kinase B (TrkB.FL) and truncated TrkB (TrkB.t1) receptors were colocalized with AChRs at NMJ and reduced TrkB expression could lead to aging-related alterations in endplate structure (Personius and Parker, 2013). TrkB.FL mRNA level increased with aging in soleus of mice from 3 to 24 months, but there was no change in TrkB.t1 mRNA level during aging. The area of TrkB immunolabeling declined with aging from 3 to 24 months and the overlap between TrkB and AChRs also declined with aging (Personius and Parker, 2013).

### Discussion on AChRs-Related Gene Expressions

Agrin-LRP4-MuSk-Rapsyn-Dok7 is the major signaling pathway driving AChRs clustering and ensuring efficient signal transduction at NMJ. Neurotrypsin could cleave Agrin and overexpression of this protease could induce endplate fragmentation, along with sarcopenia-like syndrome (Bütikofer et al., 2011). Meanwhile, the level of cleaved C-terminal Agrin fragments was higher in serum of patients with sarcopenia (Marzetti et al., 2014). LRP4 protein breakdown is increased and activated MuSk is reduced during aging (Zhao et al., 2018), which is also consistent with the declined immunolabeling area and altered distribution of TrkB and may contribute to endplate fragmentation, decreased muscle strength, and myofiber cross-section area (Kulakowski et al., 2011; Personius and Parker, 2013). This also explains the increased Sarcoglycan-α protein in old skeletal muscle, because this factor can interact with LRP4 and delay LRP4 degradation and this increase could alleviate NMJ decline in aged sarcopenia mice (Zhao et al., 2018). Therefore, advanced age is always accompanied with decreased LRP4, MuSk, and TrkB, then as a compensatory mechanism, the expression of Sarcoglycan-α is increased.

Fast-twitch muscles tend to have increased mRNA level of α and γ subunit of AChRs during aging in rats and humans (Apel et al., 2009; Mcmullen and Andrade, 2009; Aare et al., 2016; Xie et al., 2016; Soendenbroe et al., 2020). This increase precedes the appearance of sarcopenia (Ibebunjo et al., 2013). Changes of AChRs subunits mRNA level in mice need more studies to confirm. Aging preferentially changes α, γ, ε and δ mRNA and protein levels, while the difference between mRNA and protein contents may reflect a discrepancy in transcription to translation. MRFs are common myogenesis-related markers; elderly sarcopenic humans showed lower mRNA expression of MyoD and Myf5 compared with elderly non-sarcopenic humans in skeletal muscles (Brzeszczyńska et al., 2018). As mentioned before, MRFs could also regulate the expression of AChRs subunits. Aged gastrocnemius showed increased mRNA expression but decreased protein level of MRF4 (Apel et al., 2009), yet the detailed relationship between MRFs and AChRs subunits needs more investigation. Therefore, these changes of AChRs subunits in expression during the aging process may contribute to the morphological changes of post-synaptic AChRs in terms of shape, numbers, and functions, including binding efficiency shown by electrophysiological results of MEPP and EPP. No study ever compared characteristics of ACh-binding kinetics of different AChRs subunits.

**Figure 3:**
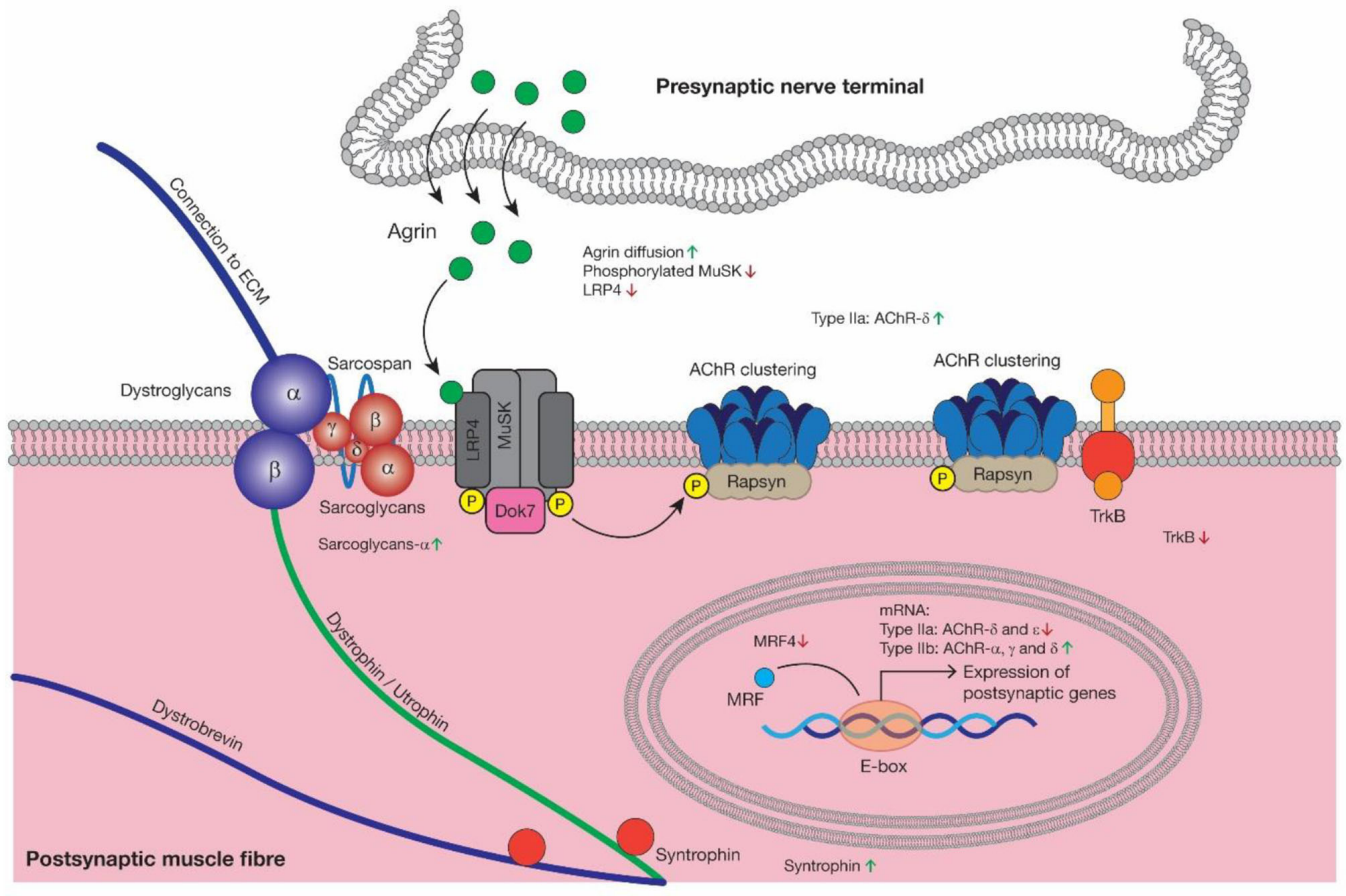
As the major signaling pathway driving AChRs clustering, Agrin diffusion, reduced MuSk activation and increased LRP4 degradation may contribute to AChRs morphological degeneration during aging, which is consistent with reduced TrkB. Then as a kind of compensatory mechanism, the expression of Sarcoglycan-α and Syntrophin was increased. MRFs could regulate AChRs gene expression and reduced MRF4 may be closely related to decreased AChR-δ and -ε in type IIa muscle fibers and increased AChRs-α, -γ, and -δ in type IIb muscle fibers at mRNA level. AChR, acetylcholine receptor; LRP4, low-density lipoprotein receptor-related protein 4; MuSk, muscle-specific kinase; Dok7, docking protein 7; Rapsyn, receptor-associated protein of the synapse; TrkB, tyrosine kinase B; MRF4, myogenic regulator factors 4.

In summary, considering the close connection between NMJ and skeletal muscles, AChRs degradation is of no doubt a main etiology of sarcopenia and investigating the detailed role of these genes in this syndrome may provide new therapeutic targets for sarcopenia. Injection of a soluble fragment of neural Agrin could improve sarcopenia phenotype in neurotrypsin-overexpressing (SARCO) mice (Hettwer et al., 2014). Increasing MuSK expression could delay denervation and improve motor function in amyotrophic lateral sclerosis (ALS) mice (Pérez-García and Burden, 2012). Rapsyn overexpression could stabilize AChRs in chronic experimental myasthenia gravis (Martínez-Martínez et al., 2007). Dok7 gene therapy has been applied to suppress motor nerve terminal degeneration at NMJ together with muscle atrophy in ALS mice and Dok7 myasthenia mice (Arimura et al., 2014; Miyoshi et al., 2017). As the most common modulating molecules of AChRs, Agrin-LRP4-MuSk-Rapsyn-Dok7 pathway has been applied in some NMJ-related disease pre-clinically. Modulation of these genes may also eventually improve symptoms of sarcopenia (Ohno et al., 2017). Also, there is currently no available medication for sarcopenia, hence investigating mechanisms of AChRs degeneration may identify a pharmaceutical target to treat sarcopenia. Changes of AChRs-related genes with aging are summarized in Figure 3.

There are several limitations in this review. Firstly, as this review only focused on aging-related changes of AChRs, some other important genes related to AChRs might not be covered, like neuregulin-1 and ColQ (Tintignac et al., 2015; Larsson et al., 2019). Secondly, only three clinical studies were included and human AChRs with increasing age were not well-discussed in this article. In addition, meta-analysis was not conducted due to data heterogeneity and only English articles were included, which may miss other available evidence.

## Conclusions

Post-synaptic AChRs of skeletal muscles undergo both morphological and functional alterations during aging and this degeneration may ultimately lead to aging-related sarcopenia. Endplate fragmentation and decreased pre- to post-synaptic ratio are commonly shown in aged skeletal muscles. As a kind of compensatory mechanism, muscles containing more MHC I fibers or MHC IIb fibers tend to have increased ACh quanta release after several stimuli with increasing age. As a result, these muscles are easier to fatigue with increasing age. To compensate for the morphological degeneration, AChRs in muscles containing more MHC IIa fibers like the diaphragm have increased availability to bind ACh, but the overall function of AChRs still reduces in old muscle fibers. Changes of AChRs area depend on the muscle type, species and the degree of muscle atrophy. AChRs subunit also experience expressional changes during aging, which is muscle type and species specific. As the key factors to form and stabilize AChRs clusters, LRP4 degradation exacerbate in old skeletal muscles along with reduced MuSk activation. Alterations of the expression levels of other related genes, such as MRFs, DGC, and TrkB may finally lead to morphological and functional adaptions of AChRs during aging. There are still several areas deserving further investigation in NMJ studies: characteristics of ACh-binding kinetics of different AChRs subunits, which can provide an approach to improve AChRs function; modulation of Agrin-LRP4-MuSk-Rapsyn-Dok7 has been applied in other NMJ-related diseases, but their therapeutic potential has not been explored in sarcopenia; human studies on NMJ are scare as well and further research is needed to identify the aging-related morphological, functional and related gene expressional alterations of AChRs in humans clinically.

## Data Availability Statement

The original contributions presented in the study are included in the article/supplementary materials, further inquiries can be directed to the corresponding author.

## Author Contributions

ZB wrote the manuscript with support from SC, RW, and W-HC. ZB and CC did the study search and selection. SC, LQ, and W-HC helped supervise the project. All authors discussed and contributed to the final manuscript.

## Conflict of Interest

The authors declare that the research was conducted in the absence of any commercial or financial relationships that could be construed as a potential conflict of interest.
